# Child sexual abuse in southern Brazil and associated factors: a population-based study

**DOI:** 10.1186/1471-2458-9-133

**Published:** 2009-05-11

**Authors:** Diego G Bassani, Lilian S Palazzo, Jorge U Béria, Luciana P Gigante, Andréia CL Figueiredo, Denise RGC Aerts, Beatriz CW Raymann

**Affiliations:** 1 Centre for Global Health Research, St Michael's Hospital, Toronto, Canada; 2 Dalla Lana School of Public Health, University of Toronto, Toronto, Canada; 3 The Hospital for Sick Children, Toronto, Canada; 4 Programa de Pós-graduação Saúde Coletiva, Universidade Luterana do Brasil, Canoas, Brazil; 5 Curso de Medicina, Universidade Luterana do Brasil, Canoas, Brazil; 6 Curso de Fonoaudiologia, Universidade Luterana do Brasil, Canoas, Brazil; 7 Universidade Federal da Bahia, Salvador, Brazil

## Abstract

**Background:**

The prevalence of child sexual abuse (CSA) in the population has been poorly described in developing countries. Population data on child sexual abuse in Brazil is very limited. This paper aims to estimate lifetime prevalence of child sexual abuse and associated factors in a representative sample of the population aged 14 and over in a city of southern Brazil.

**Methods:**

A two-stage sampling strategy was used and individuals were invited to respond to a confidential questionnaire in their households. CSA was defined as non-consensual oral-genital, genital-genital, genital-rectal, hand-genital, hand-rectal, or hand-breast contact/intercourse between ages 0 and 18. Associations between socio-demographic variables and CSA, before and after age 12, were estimated through multinomial regression.

**Results:**

Complete data were available for 1936 respondents from 1040 households. Prevalence of CSA among girls (5.6% 95%CI [4.8;7.5]) was higher than among boys (1.6% 95%CI [0.9;2.6]). Boys experienced CSA at younger ages than girls and 60% of all reported CSA happened before age 12. Physical abuse was frequently associated with CSA at younger (OR 5.6 95%CI [2.5;12.3]) and older (OR 9.4 95%CI [4.5;18.7]) ages. CSA after age 12 was associated with an increased number of sexual partners in the last 2 months.

**Conclusion:**

Results suggest that CSA takes place at young ages and is associated with physical violence, making it more likely to have serious health and developmental consequences. Except for gender, no other socio-demographic characteristic identified high-risk sub-populations.

## Background

Child sexual abuse (CSA) is one of the most stressful life events, and is associated with many adverse consequences, including physical and mental health problems, substance abuse and criminality [1]. Research in child sexual abuse has been plagued by non-representative sampling, deficient controls and limited statistical power [2]. Most available studies use school-based samples and likely underestimate child sexual abuse prevalence; on the other hand, clinic-based or out of school samples may over-estimate it. Prevalence of child sexual abuse in developed countries ranges between 6% and 60% for women and 3% and 30% for men [3,4]. This variability is a consequence of both the different definitions of child sexual abuse, measurement tools, and the populations studied [1,5]. The present study attempts to estimate the prevalence and risk factors associated with child sexual abuse in Brazil using a population-based sample.

Physical and emotional consequences of childhood sexual abuse are a cause of major concern. Several studies have identified childhood sexual abuse as a strong risk factor for psychopathology [6-8], substance dependence [7,9] and suicide [10,11]. Children that are sexually abused are not likely to report the maltreatment for fear of the consequences and, even in adulthood, the report of such events is rare due to social stigmatization [12], coercion, and as a consequence of the repression of memories [13]. The odds of disclosure of abuse vary by age, gender, and characteristics of the abuse and of the perpetrator [14]. Studies of the effects of various methodological factors on prevalence estimates of child sexual abuse revealed that male college samples report significantly higher prevalence of child sexual abuse compared to population-based samples [15]. To avoid non-representative estimates, a meta analysis of child sexual abuse prevalence studies [16] excluded samples limited to students. Such samples are non-representative of the population because they are drawn from subgroups within the community based on certain characteristics that tend to become artificially homogeneous. Reasonable estimates of the dimension of the problem and its potential burden to society can only be obtained directly in the population. Nevertheless, research on the lifetime prevalence of sexual abuse in Brazil, published from 1990 to 2007 in peer-reviewed journals, includes only 6 citations [17-22], all of which focus exclusively on school populations, with ages ranging from 15 to 24. Only one of these studies was a household survey [18], but the target population was also limited to ages ranging from 18 to 24 years. This paper aims to fill the significant gap in the Brazilian knowledge about lifetime prevalence of child sexual abuse. Using a cross-sectional survey design, this is the first study to describe the lifetime prevalence of child sexual abuse in a Brazilian population-based sample.

## Methods

This project was nested in a cross-sectional survey carried out from November 2002 to June 2003, which measured the prevalence of *hearing impairment *in the population [23].

Data were collected in the urban areas of a 306,000-inhabitant municipality from Southern Brazil. A two-stage sampling strategy was adopted. The total sample size calculated *a priori *required that 1,814 individuals be interviewed to detect a prevalence of child sexual abuse of 5% with an error level of 1% point and a 95% confidence level. Census estimates of the number of individuals per household in the municipality were used to determine the number of households that would need to be sampled to achieve the sample size. According to census data from the Brazilian Institute of Geography and Statistics for 1991 there would be an average of 3.71 persons per household in the municipality. To obtain the number of participants required for the hearing impairment study in which our study was nested, 1 040 households would need to be visited. To this end, 40 of the 391 census tracts were randomly chosen. Households were visited by 10 teams of interviewers (2 interviewers per team). For household selection, one street block was randomly selected within each of the 40 census tracts, followed by the random selection of one street corner in this block. From this corner, 26 households were systematically chosen and then visited, and all residents of the selected household, aged 14 years or more, were interviewed. Individuals who refused to participate, those still absent after a third visit, and those who were ill and could not be included in the survey are considered non-respondents. Illiterate individuals, after informed consent, had the questions read to them by one of the interviewers. The final sample includes 1,954 individuals. Further details of the study methods and field work can be found elsewhere [23].

The standardized questionnaire used in the study was pre-tested for language adequacy and logic flow in a pilot study that included a sample of 50 individuals from the same population (not included in the final sample). The sexual abuse structured questionnaire was anonymous, self-administered, and confidential. The exceptions were illiterate individuals that were assisted by the interviewer in answering the questionnaire. For confidentiality assurance, the respondent sealed the questionnaire in an envelope after its completion. Sexual abuse was defined as non-consensual oral-genital, genital-genital, genital-rectal, hand-genital, hand-rectal, or hand-breast contact or intercourse. Exposure of sexual anatomy, forced viewing of sexual anatomy, sexual intercourse and pornography were not included in the questionnaire. Information about the age of the respondent at the time when abuse took place was collected. The responses to the questionnaire were used to generate a three level outcome variable that included (1) no child sexual abuse reported, (2) report of first child sexual abuse before the age of 12 years, and (3) report of first child sexual abuse after the age of 12 years. Only information about the first episode of abuse was collected. Reports of sexual abuse after 18 years of age were included as no report of child sexual abuse. The analysis was repeated excluding individuals reporting sexual abuse after age 18 and results did not differ.

Demographic and socioeconomic variables were also collected and included age, sex, income, and education. Factors that are likely to be associated with child sexual abuse were also measured, including the prevalence of physical abuse [24], recent sexual behaviours (including use of condom in the last sexual intercourse and number of sexual partners in the last two months) [25], stressful life events in the previous 12 months (e.g. job loss, divorce, robbery) [26], self-perceived health [27], and lifetime consultation for mental health issues [28].

Data were double entered and 5% of the interviews were repeated by the field-supervisors for quality assurance. Logic and consistency checks were performed after the data were entered.

Following the descriptive analysis, the crude association between the positive reports of child sexual abuse and demographic, socioeconomic, and other factors potentially associated with the dependent variable was tested. To adequately adjust for confounders and to account for differences between child sexual abuse that is perpetrated before and after the age of 12, multinomial logistic regression was used. The modeling procedures followed a hierarchical theoretical model organized in two blocks of variables. The first block included age at interview, sex, education, and income; the second block included physical abuse, stressful life events, sexual partners in the previous two months, condom use in the most recent sexual contact, lifetime consultation for mental health issues and self-perceived health. The model was adjusted through backward elimination. Likelihood ratio tests were used to assess the heterogeneity of the estimates across the levels of the multinomial outcome (i.e. no abuse and abuse before or after age 12) compared to those obtained in models that had these same estimates constrained to be identical across the levels. Analyses were performed in Stata Version 8.0 (StataCorp, College Station, TX, 2003), accounting for the two-stage sampling design and weighting for probability of selection at the census tract and household level, as well as for non-response at household and individual levels. Sensitivity analysis was conducted by excluding the individuals reporting sexual abuse after age 18 (n = 18) but reporting no child sexual abuse.

The Ethics Committee of the Brazilian Lutheran University approved this project and all respondents signed an informed consent prior to the interviews.

## Results

A total of 1,954 individuals were interviewed in the 1,040 eligible households. In 98 households, participation in the survey was refused or no one was found after the third visit. Household losses and refusals corresponded to 9.4% of the calculated sample. In 44 of these households (losses and refusals), it was not possible to identify the number of residents. In the remaining 54 households, 615 residents were identified but did not agree to participate in the survey. Refusal was the reason for no participation stated by 410 individuals (66.7%); 189 were absent after the three consecutive visits (30.7%); and 16 were ill and could not participate (2.6%). Respondent losses and refusals were distributed across all the census tracts and corresponded to 20.3% of the eligible sample. Age and gender distribution of the final sample were compared to the census data for the corresponding tracts. The proportion of individuals 40 years and older in the sample was larger compared to the census, while the proportion of males between the ages of 20 and 39 was smaller. To account for these differences, raked sampling weights were calculated for each age and sex combination to adjust for individual level non-response and multi-stage selection probability.

Information on the presence of sexual abuse was unavailable for 18 of the respondents (0.9%), and complete data was available for 1,936 individuals that are included in the analysis. Table 1 describes the sample by age, sex, income, education, child sexual abuse, physical abuse, lifetime contacts with health professionals for mental health issues, self-perceived health status, and recent sexual behaviors.

**Table 1:** Characteristics of the survey sample by the presence and age of child sexual abuse

	**No report of child sexual abuse**			**Child sexual abuse** **(< age 12)**			**Child sexual abuse** **(age 12 to 18)**			**Total**		
	
	**n**	(w%)*	[95% CI]*	**n**	(w%)*	[95% CI]*	**n**	(w%)*	[95% CI]*	**n**	(w%)*	[95% CI]*
**Total sample**	1861	(96.1)	[95.3; 96.8]	41	(2.1)	[1.6; 2.9]	34	(1.8)	[1.2; 2.4]	1936	(100)	[n.a.]
												
**Sex **^†^												
Male	815	(46.4)	[44.5; 48.2]	8	(0.5)	[0.2; 0.9]	5	(0.3)	[0.1; 0.8]	828	(47.1)	[45.3; 48.9]
Female	1046	(49.8)	[48.1; 51.5]	33	(1.7)	[1.2; 2.3]	29	(1.5)	[1.0; 2.1]	1108	(52.9)	[51.1; 54.7]
												
**Age at interview **^†^												
Lower tertile (up to 30 y.o.)	639	(37.2)	[34.6; 39.9]	22	(1.2)	[0.8; 1.9]	14	(0.8)	[0.5; 1.2]	675	(39.2)	[36.6; 41.9]
Interm. tertile (31(46 y.o.)	575	(30.6)	[27.9; 33.3]	12	(0.6)	[0.4; 1.0]	15	(0.8)	[0.5; 1.3]	602	(31.9)	[29.2; 34.8]
Upper tertile (above 46 y.o.)	647	(28.3)	[24.9; 32.0]	7	(0.3)	[0.1; 0.7]	5	(0.2)	[0.1; 0.5]	659	(28.9)	[25.5; 32.5]
												
**Education (complete years)**												
0 to 3 years	327	(15.8)	[12.1; 20.4]	4	(0.2)	[0.1; 0.6]	7	(0.3)	[0.1; 0.8]	338	(16.3)	[13.0; 21.1]
4 to 8 years	817	(41.8)	[36.8; 47.0]	15	(0.8)	[0.5; 1.3]	17	(0.9)	[0.6; 1.4]	849	(43.5)	[38.3; 48.7]
9 to 11 years	480	(25.6)	[20.8; 31.1]	15	(0.8)	[0.5; 1.4]	8	(0.4)	[0.2; 0.8]	503	(26.8)	[21.9; 32.4]
12 or more years	237	(12.9)	[8.9; 18.3]	7	(0.4)	[0.2; 0.9]	2	(0.1)	[0.04; 0.4]	246	(13.4)	[9.2; 19.0]
												
**Income**												
Lower tertile (up to $43)	619	(31.9)	[28.4; 35.6]	18	(0.9)	[0.6; 0.2]	13	(0.7)	[0.4; 1.2]	650	(33.5)	[29.8; 37.4]
Intermediate tertile ($44–$143)	612	(30.9)	[27.7; 34.4]	12	(0.6)	[0.3; 0.1]	13	(0.6)	[0.4; 1.1]	637	(32.2)	[28.9; 35.7]
Upper tertile (above $143)	630	(33.3)	[28.1; 38.9]	11	(0.6)	[0.3; 0.1]	8	(0.4)	[0.2; 0.8]	649	(34.3)	[28.9; 40.2]
												
**Sexual partners (past 2 months)**												
None	424	(20.9)	[19.0; 22.9]	9	(0.5)	[0.3; 0.9]	6	(0.3)	[0.1; 0.6]	439	(21.6)	[19.7; 23.7]
One	1307	(68.3)	[66.0; 70.6]	29	(1.5)	[1.0; 2.2]	23	(1.2)	[0.8; 1.9]	1359	(71.1)	[68.7; 73.3]
Two or more	118	(6.9)	[5.4; 8.7]	3	(0.2)	[0.1; 0.6]	5	(0.3)	[0.1; 0.6]	126	(7.3)	[5.9; 9.2]
												
**Condom use (last intercourse)**												
Yes	549	(34.8)	[31.9; 37.9]	11	(0.7)	[0.4; 1.4]	13	(0.8)	[0.6; 1.4]	573	(36.3)	[33.2; 39.5]
No	1055	(61.5)	[58.4; 64.4]	23	(1.4)	[0.9; 2.2]	15	(0.9)	[0.4; 1.4]	1093	(63.7)	[60.5; 66.8]
												
**Stressful events past 12 months (lost job/divorce/robbery)**												
None	1415	(72.5)	[69.4; 75.4]	29	(1.5)	[1.0; 2.3]	20	(1.0)	[0.6; 1.6]	1464	(75.0)	[71.6; 78.2]
One	376	(20.6)	[17.9; 23.5]	11	(0.6)	[0.3; 1.2]	11	(0.6)	[0.3; 1.1]	398	(21.8)	[18.9; 24.9]
Two or more	53	(3.0)	[2.2; 4.0]	1	(0.05)	[0.01;0.4]	3	(0.2)	[0.1; 0.7]	57	(3.2)	[2.4; 4.3]
												
**Report of physical abuse **^†^												
No	1657	(88.5)	[87.1; 89. 8]	27	(1.5)	[1.0; 2.1]	19	(1.0)	[0.7; 1.5]	1703	(91.0)	[89.6; 92.2]
Yes	147	(7.5)	[6.3; 8. 9]	14	(0.8)	[0.4; 1.3]	15	(0.8)	[0.5; 1.3]	176	(9.0)	[7.8; 10.4]
												
**Lifetime consultation for mental health issue **^†‡^												
No	1418	(75.2)	[72.9; 77.3]	22	(1.2)	[0.8; 1.7]	23	(1.2)	[0.8; 1.8]	1463	(77.6)	[75.3; 79.6]
Yes	439	(21.0)	[18.9; 23.1]	19	(1.0)	[0.6; 1.6]	11	(0.5)	[0.3; 1.1]	469	(22.4)	[20.4; 24.7]
												
**Health perception **^†^												
Good	1192	(63.1)	[60.0; 66.1]	19	(1.0)	[0.6; 1.7]	15	(0. 8)	[0.5; 1.3]	1226	(64.9)	[61.6; 68.1]
Average/bad	667	(33.0)	[29.9; 36.3]	22	(1.1)	[0.8; 1.6]	19	(0.9)	[0.6; 1.5]	708	(35.1)	[31.9; 38.4]

* Weighted percentages and confidence intervals account for the sampling strategy. ^† ^P-value below 0.05 in the design-based chi-square test. ^‡^question included a broad term that contact with health professionals for distress, stress, emotional disturbance, psychological problems, and nervousness.

The prevalence of reported child sexual abuse in the sample was 3.9%, higher among girls (5.6%) than boys (1.6%). Over 80% of all reported first sexual abuse episodes took place before reaching 19 years of age; 63% happened before 15 years; 49% before 13 years; 27% before the children were 8 years old; and 6% before reaching 4 years of age. Among the respondents reporting being victims of sexual abuse before 19 years of age, 7.6% reported being less than 4 years old at the time; 37% were less than 8 years; 60% were less than 13 years; and 89% were less than 15 years (data available upon request).

The prevalence of self-reported sexual abuse before 12 years of age is higher among girls (1.7%) than boys (0.5%) and similarly higher among girls 12 years of age and older (1.5% vs. 0.3%). Girls experienced the majority of the total burden of child sexual abuse reported by the study participants (80% of the child sexual abuse before age 12, and 84.1% of it after age 12). While 53% of all reports of child sexual abuse for boys happened from ages 0 to 7, for girls, 33% of the child sexual abuse happened before age 8 and peaked around ages 8 to 15, when 88% of all reported abuses had already taken place (data available upon request).

The unadjusted analysis (Table 2) reveals an association between child sexual abuse before the age of 12 and female sex (OR = 3.5 [95% CI 1.7; 7.2]). Also, as the prevalence among boys decreases with age (Table 1), the strength of association of sexual abuse after age 12 with female sex increases even further (OR = 4.9 [95% CI 1.6; 15.3]).

**Table 2:** Multinomial logistic regression, factors associated with sexual abuse, before and after age12, unadjusted analysis

	**Child sexual abuse (Before age 12)**			**Child sexual abuse (Age 12 to 18)**			**p-values** **Heterogeneity test^§ ^(Likelihood ratio test)**^#^
		
	**OR**	**(95% CI)**	p-value*	**OR**	**(95% CI)**	p-value*	
**Sex**							
Male	**1.0**			**1.0**			**0.0003**
Female	**3.5**	**(1.7;7.2)**	**0.001**	**4.9**	**(1.6;15.3)**	**0.007**	**(<0.001)**
							
**Age at interview**							
Lower tertile (up to 30 y.o.)	**3.1**	**(1.2;8.1)**	**0.025**	**2.8**	**(0.9;8.4)**	**0.06**	**0.02**
Intermediate tertile (31–46 y.o.)	**1.8**	**(0.7;4.9)**	**0.23**	**3.6**	**(1.2;10.7)**	**0.02**	**(0.01)**
Upper tertile (above 46 y.o.)	**1.0**			**1.0**			
							
**Education (complete years**)							
0 to 3 years	1.0			1.0			
4 to 8 years	1.4	(0.4;4.5)	0.58	1.0	(0.4;2.5)	0.95	0.4
9 to 11 years	2.4	(0.8;6.8)	0.10	0.8	(0.3;2.3)	0.68	(0.04)
12 or more years	2.3	(0.7;7.7)	0.17	0.4	(0.1;2.0)	0.25	
							
**Income**							
Lower tertile (up to $43)	1.0			1.0			
Intermediate tertile ($44–$143)	0.7	(0.3;1.7)	0.44	1.0	(0.5;2.0)	0.99	0.5
Upper tertile (above $143)	0.6	(0.3;1.3)	0.17	0.6	(0.3;1.5)	0.29	(0.45)
							
**Sexual partners (past 2 months)**							
None	1.0			1.0			
One	1.0	(0.5;2.2)	0.9	1.3	(0.5;3.6)	0.57	0.49
Two or more	1.2	(0.3;4.3)	0.8	2.9	(0.9;9.7)	0.08	(0.43)
							
**Condom use (last intercourse)**							
Yes	0.9	(0.4;2.1)	0.82	1.5	(0.8;2.9)	0.20	0.43
No	1.0			1.0			(0.4)
							
**Stressful events past 12 months (lost job/divorce/robbery)**							
None	1.0			1.0			
One	1.5	(0.7;3.3)	0.33	2.1	(0.9;4.6)	0.07	0.13
Two or more	0.9	(0.1;5.9)	0.89	3.9	(0.9;17.3)	0.07	(0.09)
							
**Physical abuse**	**1.0**			**1.0**			**<0.001**
No	**6.2**	**(3.2;12.1)**	**<0.001**	**9.4**	**(5.2;17.1)**	**<0.001**	**(<0.001)**
Yes							
							
**Lifetime consultation for mental health issue **^†^							
No	**1.0**			1.0			**0.005**
Yes	**2.9**	**(1.5;5.5)**	**0.002**	1.6	(0.7;3.9)	0.29	**(0.002)**
							
**Health perception**							
Good	**1.0**			**1.0**			**0.009**
Average/bad	**2.1**	**(1.1;3.9)**	**0.02**	**2.2**	**(1.2;4.1)**	**0.01**	**(0.08)**

^† ^Question included a broad term that contact with health professionals for distress, stress, emotional disturbance, psychological problems, and nervousness. * Likelihood ratio test for each level of the multinomial outcome. ^§ ^Likelihood ratio test for combining alternatives. P-value corresponds to the probability that the odds ratio of a given variable with CSA before age 12 and from ages 12 to 18 is the same, indicating that both levels of the dependent variable could be collapsed. ^# ^Probability that the association of each independent variable with each of the levels of the dependent variable (child sexual abuse) is simultaneously null according to the likelihood ratio test.

Reports of sexual abuse before age 12 were more common among individuals in the younger age group (up to 30 years of age) at the date of interview. Over 50% of the child sexual abuses before the age of 12, as well as from 0 to 18 years of age, came from this age group.

The educational profile of the individuals reporting no sexual abuse, sexual abuse before the age 12, and sexual abuse from ages 12 to 18 reveals that only the group reporting sexual abuse after age 12 had a lower prevalence of abuse as years of education increased. After adjustment for age, even though the associations did not reach statistical significance, more years of education were associated with increased odds of reporting sexual abuse before 12 years of age (Table 3), in agreement with the prevalence rates presented in Table 1. The opposite was observed for reported sexual abuse after 12 years of age, but similarly, none of the estimates were statistically significant. The heterogeneity test supports these observations, revealing that the direction of the associations, across the three levels of the dependent variable, are likely to be heterogeneous (p = 0.29).

**Table 3:** Multinomial logistic regression, factors associated with sexual abuse, before and after age 12, Adjusted Odds Ratios (AOR) values.

	**Sexual abuse** **(Before age 12)**			**Sexual abuse** **(Age 12 to 18)**			**p-values** **Heterogeneity test**^§^
	
	**AOR**	**95% CI**	**p-value**	**AOR**	**95% CI**	**p-value**	**(Likelihood ratio test)**^#^
**Block I (Socio-demographic)**							
**Sex**							
Male	**1.0**			**1.0**			**0.0002**
Female	**3.6**	**(1.8; 7.3)**	**0.001**	**5.1**	**(1.6; 15.9)**	**0.006**	**(0.001)**
							
**Age at interview**							
Lower tertile (up to 30 y.o.)	**3.2**	**(1.2; 8.4)**	**0.02**	**3.0**	**(1.0; 8.7)**	**0.05**	**0.01**
Intermediate tertile (31–46 y.o.)	**1.9**	**(0.7; 5.2)**	**0.19**	**3.9**	**(1.3; 11.2)**	**0.01**	**(0.01)**
Upper tertile (above 46 y.o.)	**1.0**			**1.0**			
							
**Education (complete years)**							
*0–3 years*	*1.0*			*1.0*			
*4–8 years*	*1.2*	*(0.4; 4.2)*	*0.68*	*0.9*	*(0.3; 2.2)*	*0.64*	*0.29*
*9–11 years*	*1.9*	*(0.6; 5.4)*	*0.25*	*0.6*	*(0.2; 1.6)*	*0.22*	*(0.03)*
*12 or more years*	*1.9*	*(0.4; 7.3)*	*0.39*	*0.2*	*(0.1; 1.1)*	*0.06*	
							
**Income**							
*Lower tertile (up to $43)*	*1.0*			*1.0*			
*Intermediate tertile ($44–$143)*	*0.9*	*(0.4; 2.4)*	*0.80*	*1.5*	*(0.7; 3.2)*	*0.27*	*0.80*
*Upper tertile (above $143)*	*0.9*	*(0.3; 2.6)*	*0.88*	*1.8*	*(0.7; 4.7)*	*0.23*	*(0.45)*

**Block II**							

**Sexual partners (past 2 months)**							
*None*	*1.0*			*1.0*			
*One*	*1.3*	*(0.6; 2.7)*	*0.56*	*1.4*	*(0.6; 3.7)*	*0.43*	*0.12*
*Two or more*	*1.9*	*(0.6; 6.4)*	*0.29*	*4.5*	*(1.2; 16.2)*	*0.02*	*(0.35)*
							
**Condom use (last intercourse)**							
*Yes*	*0.8*	*(0.3; 2.2)*	*0.64*	*1.4*	*(0.7; 2.9)*	*0.35*	*0.63*
*No*	*1.0*			*1.0*			*(0.5)*
							
**Stressfull events past 12 months (lost job/divorce/robbery)**							
*None*	*1.0*			*1.0*			
*One*	*1.1*	*(0.4; 2.7)*	*0.84*	*1.4*	*(0.5; 3.8)*	*0.61*	*0.85*
*Two or more*	*0.4*	*(0.1; 3.6)*	*0.43*	*1.4*	*(0.2; 9.5)*	*0.34*	*(0.08)*
							
**Physical abuse**							
No	**1.0**			**1.0**			**< 0.001**
Yes	**5.6**	**(2.5; 12.3)**	**< 0.001**	**9.4**	**(4.5; 18.7)**	**< 0.001**	**(< 0.001)**
							
**Lifetime consultation for mental health issue**							
No	**1.0**			1.0			**0.10**
Yes	**2.2**	**(1.1; 4.6)**	**0.04**	0.9	(0.3; 2.6)	0.84	**(0.003)**
							
**Health perception**							
Good	**1.0**			**1.0**			**0.04**
Average/bad	**2.0**	**(1.1; 3.8)**	**0.03**	**1.9**	**(1.1; 3.6)**	**0.04**	**(0.005)**

Reference category is 'no abuse'. Hierarchical model with backwards elimination (final model n = 1874)

Note: Bolded variables are in the final model and are adjusted to each other. Estimates in italics were obtained from each variable's last entry in the model, before it was removed from the model (backwards elimination). Variables from subsequent levels are adjusted for bolded variables from the same level and from preceding levels.^§ ^Likelihood ratio test for combining alternatives. P-value corresponds to the probability that the odds ratio of a given variable with CSA before age 12 and from ages 12 to 18 is the same, indicating that both levels of the dependent variable could be collapsed. ^# ^Probability that the association of each independent variable with each of the levels of the dependent variable (child sexual abuse) is simultaneously null according to the likelihood ratio test.

Although 75% of the children that were sexually abused before 12 years of age belonged to the lower income group, income was not associated with either category of child sexual abuse in the multinomial model.

In the crude analysis, a strong association between physical and child sexual abuse before the age of 12 was observed (OR 5.6 95%CI [2.5;12.3]), and this association was stronger when the reported abuse happened after the age of 12 (OR 95% 9.4 95%CI [4.5;18.7]). This pattern was maintained after adjustment for the other variables in the model (Table 3).

A trend towards reporting occurrence of physical and sexual abuse at the same age was observed among boys but not girls (data not shown). Overall, no specific age or gender pattern of co-occurrence of sexual and physical abuse could be observed. The amount of missing information about the age of physical abuse among boys compromises more elaborate analysis.

Lifetime contacts with health professionals for mental health concerns were more prevalent among those reporting sexual abuse before age 12. This variable was associated with child sexual abuse before age 12, before and after adjustment, but not for child sexual abuse after age 12.

Individuals that experienced child sexual abuse at any age were more likely to perceive their health as average or bad compared to individuals reporting no child sexual abuse experiences. The odds ratio for self-perceived bad or average health was 2.0 (95% CI 1.1; 3.8) before and 1.9 (95% CI 1.1; 3.6) after 12 years of age.

Although a trend towards a larger number of sexual partners in the previous 2 months can be suspected for both age groups, it only achieved statistical significance when the abuse took place after 12 years of age (OR for two or more sexual partners in the previous 2 months = 4.5 [95% CI 1.2; 16.2]).

Because the sample includes 202 individuals with ages between 14 and 18 – 96% of which are still at risk of experiencing child sexual abuse as defined in the present study – sensitivity analysis was performed to verify the direction and strength of the estimates in the absence of these respondents. No changes (defined as a change in the adjusted odds ratio that was larger than 5% of the original estimate) were observed and therefore all individuals were included in the analysis.

## Discussion

To the best of the authors' knowledge, this is the first anonymous population-based study estimating the prevalence of child sexual abuse in a comprehensive sample from Brazil. The data reveal that the prevalence of self-reported sexual abuse at very young ages (before 12 years) is more than three times higher among girls (1.7%) compared to boys (0.5%) and almost five times higher among girls (1.5% vs. 0.3%) after 12 years of age. Reports from Brazil [18] support our findings, indicating that girls are more often sexually abused in childhood compared to boys. In a study estimating the contribution of selected risk factors for the global burden of disease, child sexual abuse was found to be the only childhood exposure that has a differential – and larger – contribution to health loss for girls compared to boys [29].

Physical abuse was strongly associated with child sexual abuse in the sample. Previous analysis of this survey focused on lifetime physical abuse and indicated high prevalence (9.7%) and suggests an increased need for health care – as a consequence of physical violence – in this population [30]. Results from studies conducted in the US reveal unsettling trends for the occurrence of multiple episodes of victimization once the first episode has taken place [31] and a consequent increase in risk for mental disorders [31,32]. In addition, children exposed to various kinds of abuse are more symptomatic than children exposed to a similar number of episodes of the same kind [33].

There was a higher prevalence of self-reported lifetime contact with health professionals for mental health reasons among individuals abused before 12 years of age. Adjustment for confounding variables still revealed an association, suggesting there may be no difference in contacts with professionals for mental health reasons when comparing individuals abused after age 12 to those reporting no abuse (p-value for heterogeneity = 0.11). Similar findings were observed in a cohort study of Australian sexually abused children; estimates of the use of mental health services were nearly four times higher among the abused group, compared to population controls [34]. The lack of such association for those abused after age 12 may indicate that the psychological consequences of the abuse are more severe when it takes place at earlier ages [4,35]. Other factors such as the child's developmental stage, the chronicity of the abusive acts, the child's resilience, and the child's relationship with the perpetrator may also influence the psychological and behavioral consequences of the abuse [1] reflected in the increased contact with health professionals for mental health issues.

In light of these results, it is likely that interventions to reduce the prevalence of child sexual abuse should focus on the population as a whole, since it appears that, barring gender differences, none of the demographic characteristics studied permit the easy identification of high risk sub-groups. Additionally, attention should be devoted to the prevention of child sexual abuse among young girls.

Following the recent call for better information on prevalence and factors associated with child sexual abuse [2], we expect that this work will reach policy tables and instigate action. Child sexual abuse alone is responsible for about 1% of the global burden of disease, but is likely to be a risk factor for several other diseases – including alcohol and illicit drugs use, mental disorders, and sexually transmitted diseases – which combined, are responsible for over 20% of the global burden [29].

Identification of abuse is an important means of preventing repetition. Stimulating the children's competency in recognizing abusive situations, discerning between appropriate and inappropriate contacts, and about the importance of disclosing the abuse, may be part of the solution to the problem [36], but the high concentration of CSA at very young ages suggests complex barriers to the implementation of such strategies. As the epidemiology of child sexual abuse becomes better understood, it is important to prepare for the planning and evaluation of preventive interventions. Development of effective treatment of sexually abused children, and of child abusers, is essential for future reduction of the rates of child sexual abuse.

### Study Limitations

The prevalence estimates presented here may be underestimated due to the use of a short questionnaire that defined sexual abuse and asked about the occurrence of such event and the age when it first happened. Nevertheless, it has been suggested that even after a series of 19 screening questions, 12% of female victims of sexual abuse will not disclose the event [16]. In addition, the prevalence estimates presented here refer exclusively to reports of contact sexual abuse. It is likely that the observed prevalence of 3.9% is not accounting for a much larger prevalence of sexual abuse without contact, had it been measured. Data from Switzerland and Australia suggest that sexually abusive events without contact are much more prevalent than ones with contact [37,38], with similar patterns likely to be present in the current sample. Also, surveys are prone to underestimation of true prevalence due to underreporting and recall bias, especially in the field of child sexual abuse [39] and the observed association between age at interview and child sexual abuse may be a consequence of such bias. The percentage of individuals unable to recall sexual abuse events has been reported to be as high as 38% after 17 years from the event [40]. The possibility of such bias justifies the inclusion of the variable age at interview in the final model.

The cross-sectional nature of the study, though, does not allow for causal inferences and limits interpretation of the estimated associations. In addition, this sample was drawn from a low-income urban area of southern Brazil, and it may not be representative of rates observed among higher income groups, in other parts of the country or among rural communities. Refusals and losses were more common among males, and because the prevalence of child sexual abuse is lower among them, overall prevalence could be artificially inflated. To address this issue raked sampling weights were used to adjust for individual level non-response and multi-stage selection probability.

The very low income of the population in the present study may have hindered the emergence of an association of income with child sexual abuse due to the low variability of income. However, the lack of association between child sexual abuse, income, and education is supported by results from another epidemiological study [41] and it has been suggested that for child sexual abuse screening purposes, no identifiable demographic or family characteristics would allow one to exclude the possibility that a child was sexually abused [42].

The observed association of age of the respondent at interview and child sexual abuse may indicate an increase in its prevalence among younger age groups but may also be an artifact of recall and survivorship biases. More detailed longitudinal methods are necessary to clarify this issue.

The survey did not identify the relationship of the victim with the perpetrator but this information would have added richness to the estimates. Data from other jurisdictions indicate that over two-thirds of child sexual abuse perpetrators are family members or acquaintances [43]. The fact that the perpetrators are likely to be the children's caretakers adds a challenging dimension to prevention of child sexual abuse and to its study in the population [1]. There is also evidence that abuse within a family is likely to be more persistent and frequent than extra-familial abuse [44].

Finally, face-to-face interviews were conducted with respondents that had less than 4 years of education (no more than 15.8% of the sample) but an analysis of the influence of mode of interview in the results was not possible since the questionnaires were not identified. The literature suggests there is no agreement about the consequences of mode of interview in the estimates of prevalence of child sexual abuse [45,46] and Bolen and Scannapiecco [16] observed in their meta analysis that mode of interview did not affect prevalence estimates for males or females.

## Conclusion

Results suggest that Child Sexual Abuse in Brazil happens at young ages and is usually associated with physical violence. This adverse event is likely to have serious health and developmental consequences. Except for gender, no other socio-demographic characteristic identified high-risk sub-populations, making it difficult to single-out at-risk groups for prevention and indicating preventive strategies should be widespread.

## Competing interests

The authors declare that they have no competing interests.

## Authors' contributions

DB conducted the analysis planning, execution, and lead the preparation of the manuscript. JB planned and coordinated the study and contributed to the report. LP participated in the study planning and contributed to the report. LG participated in the study planning, data collection, elaboration and preparation of the dataset, and contributed to the report. AF participated in the study planning, data collection, elaboration and preparation of the dataset, and contributed to the report. DA participated in the study planning and contributed to the report. BR participated in the study planning and contributed to the report.

## Pre-publication history

The pre-publication history for this paper can be accessed here:


